# Effects of saturation flash protocols on photosynthetic induction and assessment of mesophyll conductance and maximum carboxylation rate sensitivity to parameter inputs in *Ficus carica*

**DOI:** 10.3389/fpls.2026.1760513

**Published:** 2026-03-17

**Authors:** Wei Jin, Guoyin Li, Jinping Wang

**Affiliations:** 1 College of Life Science and Agronomy, Zhoukou Normal University, Zhoukou, China; 2 Department of Ultrasonic Medicine, Beijing Chest Hospital, Capital Medical University & Beijing Tuberculosis and Thoracic Tumor Research Institute, Beijing, China

**Keywords:** biochemical capacity, chlorophyll fluorescence, mesophyll conductance, photosynthetic induction, photosynthetic limitations

## Abstract

Improving photosynthetic efficiency during the induction phase is a key strategy for enhancing plant productivity. A critical prerequisite for achieving this goal is identifying the limitation factors that affect photosynthesis during induction. While stomatal conductance to water vapor (g_sw_) can be quantified using gas exchange measurements, the quantification of mesophyll conductance (g_m_) and maximum carboxylation rate (*V_cmax_*) remains uncertain and underexplored. This study, using *Ficus carica* as plant material, aims to investigate whether saturation flashes influence the photosynthetic induction process and assess the sensitivity of g_m_, *V_cmax_*, and photosynthetic limitation analysis to key photosynthetic parameters. The results revealed no significant differences in the dynamics of photosynthesis and stomatal conductance between treatments with and without saturation flashes, indicating that saturation flashes did not interfere with the photosynthetic induction process. Further analysis of the photosynthetic induction process showed that g_m_ and *V_cmax_* exhibited a gradual increase during induction. Moreover, sensitivity analysis demonstrated that variations in the leaf absorptance parameter (αβ) and CO_2_ compensation point (Γ^*^) significantly impacted the estimation of g_m_ and *V_cmax_*, while changes in the Rubisco affinity constant (*K_m_*) also notably influenced *V_cmax_*. Finally, photosynthetic limitation analysis revealed that stomatal limitation (SL) was the primary factor limiting photosynthesis in *Ficus carica*, accounting for 41.4% of the total limitation, followed by mesophyll conductance limitation (MCL) and biochemical limitation (BL), which accounted for 30.9% and 27.7%, respectively. Statistical analysis revealed no significant changes in the overall pattern of photosynthetic limitations across different sensitivity analysis scenarios. Overall, this study demonstrates that saturation flashes do not interfere with photosynthetic induction in *Ficus carica*, while changes in αβ, Γ^*^, and *K_m_* affect the estimation of g_m_ and *V_cmax_* but have no significant impact on the estimation of photosynthetic limitations. This study provides a theoretical basis for improving photosynthetic efficiency during the induction phase in *Ficus carica* and enhances our understanding of photosynthetic induction measurement and calculation methods.

## 1 Introduction

Enhancing photosynthetic efficiency is widely regarded as an effective means of increasing plant productivity (Zhu et al., 2008). Numerous studies have examined the relationship between productivity and photosynthetic traits assessed under steady-state light conditions. Although a positive correlation has been observed in some cases (Medrano et al., 2003; Gaju et al., 2016), other studies report no significant association (Driever et al., 2014; Sommer et al., 2024). In natural environments, however, light intensity fluctuates continuously, rendering steady-state traits insufficient for characterizing actual photosynthetic performance. During transitions from low to high light, photosynthetic rates rise progressively through a process known as photosynthetic induction (Pearcy, 1990). Because of the delay inherent to this process, instantaneous photosynthetic efficiency under sudden high light is lower than the steady-state efficiency achievable under the same irradiance, with reported losses of up to 21% (Taylor and Long, 2017). Therefore, improving photosynthetic efficiency in the induction phase, especially by accelerating the attainment of steady state, offers a promising strategy for increasing overall plant productivity.

Current mechanistic understanding indicates that photosynthetic induction following a low-to-high light transition is governed by three major processes: (i) the induction kinetics of photosynthetic electron transport in the thylakoid membrane (Yamori et al., 2016), (ii) the activation of Calvin–Benson cycle enzymes, especially Rubisco (Pearcy and Seemann, 1990; Yamori et al., 2012), and (iii) CO_2_ diffusion along the leaf–chloroplast pathway (Pearcy, 1990). The CO_2_ diffusion pathway comprises two sequential conductance: diffusion from the leaf surface into the intercellular airspaces through stomata, defined as stomatal conductance (g_s_), and diffusion from the intercellular spaces to the chloroplast stroma, referred to as mesophyll conductance (g_m_) (Evans et al., 1986). Over the past decade, substantial research has focused on stomatal kinetics and their effects on photosynthetic induction (Lawson and Blatt, 2014; McAusland et al., 2016; Taylor and Long, 2017). In contrast, g_m_ was traditionally considered a minor limiting factor (Lawson and Blatt, 2014). However, recent evidence from stable-isotope methods indicates that g_m_ can impose considerable limitations on photosynthetic induction, contributing up to approximately 35% of the total limitation (Liu et al., 2022).

Identifying the key limiting factors during photosynthetic induction is therefore critical for enhancing photosynthetic efficiency. Quantifying dynamic changes in g_m_ has been shown to be essential for accurate limitation analyses, as neglecting g_m_ dynamics may lead to misidentification of the dominant limiting processes (Pan et al., 2025). Although stable-isotope approaches provide robust estimates of g_m_, they require expensive laser spectrometers, complex measurement procedures, and substantial theoretical knowledge (Ubierna et al., 2018). Consequently, many studies have employed chlorophyll fluorescence–based techniques as an alternative (Sun et al., 2022a, Sun et al., 2022b; Sun et al., 2023; Wang et al., 2023). Yet these approaches often overlook a fundamental prerequisite: whether the saturating multiphase flashes used during fluorescence measurements affect the leaf photosynthetic induction state. This verification is essential before fluorescence-based approaches can be applied quantitatively. Furthermore, the computation of g_m_ depends on several parameters, including the electron transport rate (*J*) and the CO_2_ compensation point in the presence of mitochondrial respiration (Γ^*^), that are frequently treated as fixed values rather than being empirically determined (Sun et al., 2022a, Sun et al., 2022b; Sun et al., 2023; Wang et al., 2023). Such simplification is already recognized as inappropriate under steady-state conditions, where parameter calibration is required (Xiong et al., 2015). Thus, parameter calibration during induction, coupled with sensitivity analyses, is urgently needed to clarify how parameter variability influences g_m_ estimation.

Referring to the studies of Yamori et al. (2012, Yamori et al., 2016), RuBP regeneration typically completes within the first 1–2 minutes of photosynthetic induction, while Rubisco activation requires a longer duration. Therefore, biochemical limitations during photosynthetic induction are typically attributed to Rubisco limitation, rather than RuBP-regeneration limitation (Liu et al., 2022; Pan et al., 2025). Current quantification of Rubisco activation typically relies on the Farquhar model (Farquhar et al., 1980), which estimates Rubisco carboxylation capacity (*V_cmax_*) from net photosynthesis, chloroplastic CO_2_ concentration (C_c_), and Rubisco kinetic constants. Because g_m_ estimation is highly sensitive to parameter variation, changes in these inputs alter g_m_, which in turn affects C_c_ and consequently propagates uncertainty into *V_cmax_* estimates. To date, however, the influence of g_m_-related parameter variability on *V_cmax_* during the induction process has not been examined. Moreover, many studies rely on the Michaelis-Menten constant (*K_m_*) values for Rubisco that were determined in other species rather than measured in the plant material under investigation (Xiong et al., 2015; Pan et al., 2025). Substantial interspecific variation in Rubisco kinetic parameters has been documented (Orr et al., 2016), yet its impact on *V_cmax_* estimation during induction remains unexplored. If g_m_ varies with parameters such as the leaf’s light absorption rate (α), the proportion of light quanta absorbed by Photosystem II (β), and Γ^*^, and *V_cmax_* varies wit α, β, Γ^*^, and *K_m_*, such parameter uncertainties may alter limitation analyses and ultimately influence the identification of the key limiting factors during photosynthetic induction.

To address these gaps, we used *Ficus carica* as a model system. We first evaluated whether the saturating flashes applied during chlorophyll fluorescence measurements affect photosynthetic induction traits. We then performed a sensitivity analysis to determine how parameter variation influences the estimation of g_m_, *V_cmax_*, and photosynthetic limitation values. Specifically, this study aimed to: (1) standardize the application of chlorophyll fluorescence techniques for photosynthetic induction measurements, and (2) identify the major limiting factors governing photosynthetic induction in *Ficus carica*.

## 2 Materials and methods

### 2.1 Plant materials and growth conditions

*Ficus carica* Linn. seedlings exhibiting consistent growth and having four true leaves were purchased from a nursery in Zhoukou. They were immediately transplanted into 5 L containers with substrate and slow-release fertilizer to provide the necessary nutrients for growth at Zhoukou Normal University in March. The seedlings were cultivated outdoors with daily irrigation. During March, the average temperature in Zhoukou was 22 °C, with a relative humidity of 55%. Prior to photosynthetic measurements, the seedlings were transferred to a greenhouse for acclimation for 3 days. The greenhouse conditions were set with a light intensity of 1000 mmol m^-^² s^-^¹, an air humidity of 60%, a daytime temperature of 25 °C, a nighttime temperature of 20 °C, and a photoperiod of 14 hours during the day and 10 hours at night humidity of 60%.

### 2.2 Experimental design and measurement protocol

To examine the effect of saturation flashes during fluorescence measurements on the photosynthetic induction process, four fully expanded leaves of *Ficus carica* Linn. were selected, the following experimental design was employed: Gas exchange measurements during photosynthetic induction were initially performed using a LI-6800 system equipped with a fluorescence leaf chamber without using saturation flashes. To avoid potential order or acclimation effects, photosynthetic induction measurements with and without saturation flashes were performed on the same set of leaves. To ensure this, measurements without saturation flashes were conducted on the first day, followed by measurements with saturation flashes on the second day. This approach was adopted to minimize any confounding influence of measurement order on the results. Light levels were set at 100 µmol photons m^-^² s^-^¹ (low light) and 1200 µmol photons m^-^² s^-^¹ (high light), with a CO_2_ concentration of 400 µmol mol^-^¹, a temperature of 25 °C, humidity at 60%, and a gas flow rate of 500 µmol s^-^¹. The leaves were acclimated under low light for 30 minutes, and gas exchange parameters (net photosynthetic rate, A; and stomatal conductance to water vapor, gsw) were recorded every minute. Subsequently, both gas exchange and fluorescence parameters, including steady-state fluorescence (Fs) and maximum fluorescence (Fm'), were measured using the fluorescence leaf chamber. For this experiment, saturation flashes were applied delivered using a leaf multiphase flash (MPF) fluorometer chamber (6800-01A; LI-COR, Inc.). The MPF settings were optimized as follows: a flash intensity of 8500 µmol m^-^² s^-^¹, measuring beam intensity ranging from 1 to 2 µmol m^-^² s^-^¹, a 60% reduction in flash intensity during the second phase, and durations of 0.3, 0.7, and 0.4 seconds for the three consecutive flash phases. The flashes were applied at intervals between 61 and 77 seconds. Using these fluorescence data, the quantum efficiency of Photosystem II (ΦPSII) was calculated according to the following formula:

(1) $$\begin{array}{c} {\text{Φ}}_{\text{PSII}}\;=\;\frac{{\text{F}}_{\text{m}}\prime -{\text{F}}_{\text{s}}}{{\text{F}}_{\text{m}}\prime } \end{array}$$

The linear electron transport rate (*J*) through Photosystem II is determined using the following formula:

(2) $$\text{J}={\text{Φ}}_{\text{PSII}}*\text{PPFD}*\text{αβ}$$

In these equations, α represents the leaf’s light absorption rate, and β denotes the proportion of light quanta absorbed by Photosystem II. The method proposed by Yin et al. (2009) was employed, where a standard steady-state light response curve is obtained under 2% O_2_ conditions. Regression analysis is then performed on the linear relationship between Φ_PSII_ × PPFD/4 and *A* to determine the parameters α and β.

Following the variable J method of Harley et al. (1992), the relationship between mesophyll conductance and J is expressed as:

(3) $$\begin{array}{c} g_{m}=\frac{A}{C_{i}-\frac{{\text{Γ}}^{*}(J+8*(A+{\text{R}}_{\text{d}})}{J-4*(A+{\text{R}}_{\text{d}})}} \end{array}$$

In this context, C_i_ refers to the intercellular CO_2_ concentration, Γ* is the CO_2_ compensation point during mitochondrial respiration, and Rd represents the mitochondrial respiration rate. Γ* is further expressed as:

(4) $$\begin{array}{c} \Gamma^{*}=C_{i}^{*}+\frac{R_{d}}{g_{m}} \end{array}$$

Here, C_i_^*^ represents the apparent CO_2_ compensation point. R_d_ and C_i_^*^ are determined using the Laisk method (Brooks and Farquhar, 1985), which involves plotting the *A*/C_i_ curve at three different sub-saturating light levels (150, 300, and 600 μmol m^−2^ s^−1^). The intersection of these curves provides the values for C_i_^*^ and R_d_, respectively. Using formulas 3 and 4, the unknowns g_m_ and Γ^*^ can then be solved.

### 2.3 Quantification of limitations during photosynthetic induction

Total deviation of *A* from its steady-state value (d*A*_calc_) was is primarily influenced by biochemical, g_m_, and g_s_ factors as Liu et al. (2022):

(5) $$\begin{array}{c} \text{d}A_{\text{calc}}=\text{d}A_{\text{biochem}}+\text{d}A_{\text{mesophyll}}+\text{d}A_{\text{stom}} \end{array}$$

Here, d*A*_biochem_, d*A*_mesophyll_, and d*A*_stom_ represent the biochemical, mesophyll, and stomatal contributions to d*A*_calc_, respectively. d*A*_biochem_ can be further expressed as:

(6) $$\begin{array}{c} \text{d}A_{\text{biochem}}=\frac{\partial A}{\partial V_{c\max}}dV_{cmax} \end{array}$$

Meanwhile, dAbiochem and dAstom are represented as:

(7) $$\begin{array}{c} \text{d}A_{\text{mesophyll}}=\frac{\partial A}{\partial {\text{g}}_{\text{m}}}d{\text{g}}_{\text{m}} \end{array}$$

(8) $$\begin{array}{c} \text{d}A_{\text{stom}}=\frac{\partial \text{A}}{\partial {\text{g}}_{\text{sc}}}\text{d}{\text{g}}_{\text{sc}} \end{array}$$

In these equations, d*V_cmax_*, dg_m_, and dg_sc_ represent the differences between the real-time measurements of *V_cmax_*, g_m_, and g_sc_ at any given time point during the photosynthetic induction process and their values at steady-state conditions. g_sc_ refers to stomatal conductance to CO_2_ (g_sc_ = g_sw_/1.6).

The Rubisco-limited *A* can be expressed as:

(9) $$\begin{array}{c} A=\frac{V_{cmax}\left({\text{C}}_{\text{c}}-{\text{Γ}}^{*}\right)}{{\text{C}}_{\text{c}}+K_{m}}-{\text{R}}_{\text{d}} \end{array}$$

Where Km is the Michaelis-Menten constant of Rubisco under 21% O2, which is 541.9 μmol mol^-^¹ at 25 °C in Arabidopsis (Walker et al., 2013). By combining Equation 10 with the following CO2 diffusion equation:

(10) $$\begin{array}{c} {\text{C}}_{\text{c}}={\text{C}}_{\text{a}}-\frac{A}{{\text{g}}_{\text{sc}}}-\frac{A}{{\text{g}}_{\text{m}}} \end{array}$$

Equations 9, 10 can be rearranged as:

(11) $$\left(\frac{1}{g_{sc}}+\frac{1}{g_{m}}\right)A^{2}-\left[\left(V_{c\max}-R_{d}\right)\left(\frac{1}{g_{sc}}+\frac{1}{g_{m}}\right)+\left(C_{a}+K_{m}\right)\right]A+V_{\text{c},\text{max}}\left({\text{C}}_{\text{a}}-\Gamma^{*}\right)-R_{\text{d}}\left(C_{\text{a}}+K_{\text{m}}\right)=0$$

According to Equation 11, the derivatives of Vcmax, gm, and gsc with respect to A were calculated:

(12) $$\begin{array}{c} \frac{\partial A}{\partial V_{\text{c}\max}}=\frac{\left(C_{\text{a}}-\Gamma^{*}\right)-A\left(\frac{1}{{\text{g}}_{\text{sc}}}+\frac{1}{{\text{g}}_{\text{m}}}\right)}{\left(V_{\text{c}\max}-R_{d}\right)\left(\frac{1}{{\text{g}}_{\text{sc}}}+\frac{1}{{\text{g}}_{\text{m}}}\right)+\left(C_{\text{a}}+K_{\text{m}}\right)-2\left(\frac{1}{{\text{g}}_{\text{sc}}}+\frac{1}{{\text{g}}_{\text{m}}}\right)A} \end{array}$$

(13) $$\begin{array}{c} \frac{\partial A}{\partial {\text{g}}_{\text{m}}}=\frac{\frac{A}{{\text{g}}_{m}^{2}}\left(\left(V_{\text{c}\max}-R_{d}\right)-A\right)}{\left(V_{\text{c}\max}-R_{d}\right)\left(\frac{1}{{\text{g}}_{\text{sc}}}+\frac{1}{{\text{g}}_{\text{m}}}\right)+\left(C_{\text{a}}+K_{\text{m}}\right)-2\left(\frac{1}{{\text{g}}_{\text{sc}}}+\frac{1}{{\text{g}}_{\text{m}}}\right)A} \end{array}$$

(14) $$\begin{array}{c} \frac{\partial A}{\partial {\text{g}}_{\text{sc}}}=\frac{\frac{A}{{\text{g}}_{\text{sc}}^{2}}\left(\left(V_{\text{c}\max}-R_{d}\right)-A\right)}{\left(V_{\text{c}\max}-R_{d}\right)\left(\frac{1}{{\text{g}}_{\text{sc}}}+\frac{1}{{\text{g}}_{\text{m}}}\right)+\left(C_{\text{a}}+K_{\text{m}}\right)-2\left(\frac{1}{{\text{g}}_{\text{sc}}}+\frac{1}{{\text{g}}_{\text{m}}}\right)A} \end{array}$$

At any time point (t), the value of V_cmax_ (V_cmax_(t)) can be calculated using the following equation (Deans et al., 2019a):

(15) $$V_{\text{c}\max}(\text{t})=\frac{\left(A(\text{t})+R_{\text{d}})({\text{C}}_{\text{c}}(\text{t})+K_{m}\right)}{{\text{C}}_{\text{c}}(\text{t})-{\text{Γ}}^{*}}$$

Throughout the entire photosynthetic induction process, the relative limitations on *V_cmax_* (BL), g_m_ (MCL), and g_sc_ (SL) are calculated as the time integral of the respective terms:

(16) $$\begin{array}{c} \text{BL}\;\text{or}\;\text{MCL}\;\text{or}\;\text{SL}=\frac{\int \text{d}A_{\text{biochem}/\text{mesophyll}/\text{stom}}\text{d}t}{\int \text{d}A_{\text{calc}}\text{dt}} \end{array}$$

### 2.4 Sensitivity analysis of g_m_ and *V_cmax_* and their impact on photosynthetic limitation analysis

During the photosynthetic induction process, g_m_ is calculated according to Equation 3. However, many photosynthetic induction studies typically fix the αβ value for *J* calculation at 0.42-0.45, and Γ^*^ is commonly set at 40 μmol mol^-1^ (Sun et al., 2022a, Sun et al., 2022b). It is important to note, however, that Γ* has not always been treated as a constant in steady-state photosynthetic studies (Sharkey et al., 2007; Xiong et al., 2015). While a fixed value is necessary for conducting a sensitivity analysis, we varied the αβ and Γ^*^ values by ±10%, based on measured values, to assess their effect on g_m_ in this study. Since changes in g_m_ lead to variations in C_c_, and according to Equation 15, changes in C_c_ subsequently affect the calculation of *V_cmax_*, we also evaluated how these parameters variations influence *V_cmax_*. Additionally, the *K_m_* value used in the *V_cmax_* calculation is fixed in previous photosynthetic induction study (Liu et al., 2022), therefore the 541.9 μmol mol^-1^ used in this study following Liu et al. (2022). It is important to note, however, that *K_m_* has not always been treated as a constant in steady-state photosynthetic studies (Bernacchi et al., 2002; Diao et al., 2025). While a fixed value is necessary for conducting a sensitivity analysis, we similarly varied the *V_cmax_* value by ±10% to examine its impact on *V_cmax_*. Given that changes in g_m_ and *V_cmax_*, as described by Equations 12, 13, 14, can affect the calculation of photosynthetic limitation values, we analyzed the effect of variations in these three parameters on photosynthetic limitation. Specifically, we recalculated the values of SL, MCL, and BL by substituting the updated *V_cmax_* and g_m_ values into Equations 12–15.

### 2.5 Induction kinetics of photosynthetic parameters

The induction kinetics of photosynthetic parameters can be represented by an exponential model as Mott and Woodrow (2000):

(17) $$\begin{array}{c} F\left(\text{t}\right)=F_{\text{f}}+(F_{\text{i}}-F_{\text{f}})e^{\frac{-\text{t}}{\text{τ}}} \end{array}$$

Where Fi and Ff represent the minimum and maximum values of photosynthetic parameters (A, and gsw) during the induction process, and t is the time constant for the parameter. To further calculate the time to reach 90% of the maximum value:

(18) $${\text{t}}_{90}=-\tau \ln(0.01)$$

Assimilated CO2 during photosynthetic induction can be calculated as follows:

(19) $$\begin{array}{c} \text{C}\;\text{gain}=\int_{0}^{t}A\left(t\right)dt \end{array}$$

Carbon loss ratio, the percentage of potential CO2 uptake forgone due to the lower rates through induction compared with steady state, was calculated as:

(20) $$\begin{array}{c} \text{Carbon}\;\text{loss}\;\text{ratio}=\frac{A_{\text{f}}*\text{t}-\text{C}\;\text{gain}}{A_{\text{f}}}*100 \end{array}$$

Where *A*_f_ represents the maximum values of photosynthetic parameters during photosynthetic induction.

### 2.6 Statistical analysis

A t-test was used to compare the parameters *A* and g_sw_ under low light (e.g. *A*_i_, and g_swi_), as well as *A*_f_, g_swf_ after high light exposure, and response speed of *A* and g_sw_ (e.g. t_A90_, and t_gsw90_) between treatments with and without saturation flashes. If no significant differences were found between these six parameters, it would indicate that saturation flashes do not affect the photosynthetic induction state. The limitation analysis values were analyzed using *ANOVA* to compare whether there were significant differences in SL, MCL, and BL within the same sensitivity scenario, as well as whether significant differences existed in each limiting component across different sensitivity scenarios. *Post-hoc* multiple comparisons were performed using LSD (*P* < 0.05). All statistical analyses were conducted using SPSS 21.0 (IBM Corp., Armonk, NY, USA).

## 3 Results

### 3.1 Assimilation and stomatal induction dynamics with and without saturation flashes during induction

**Figure 1:**
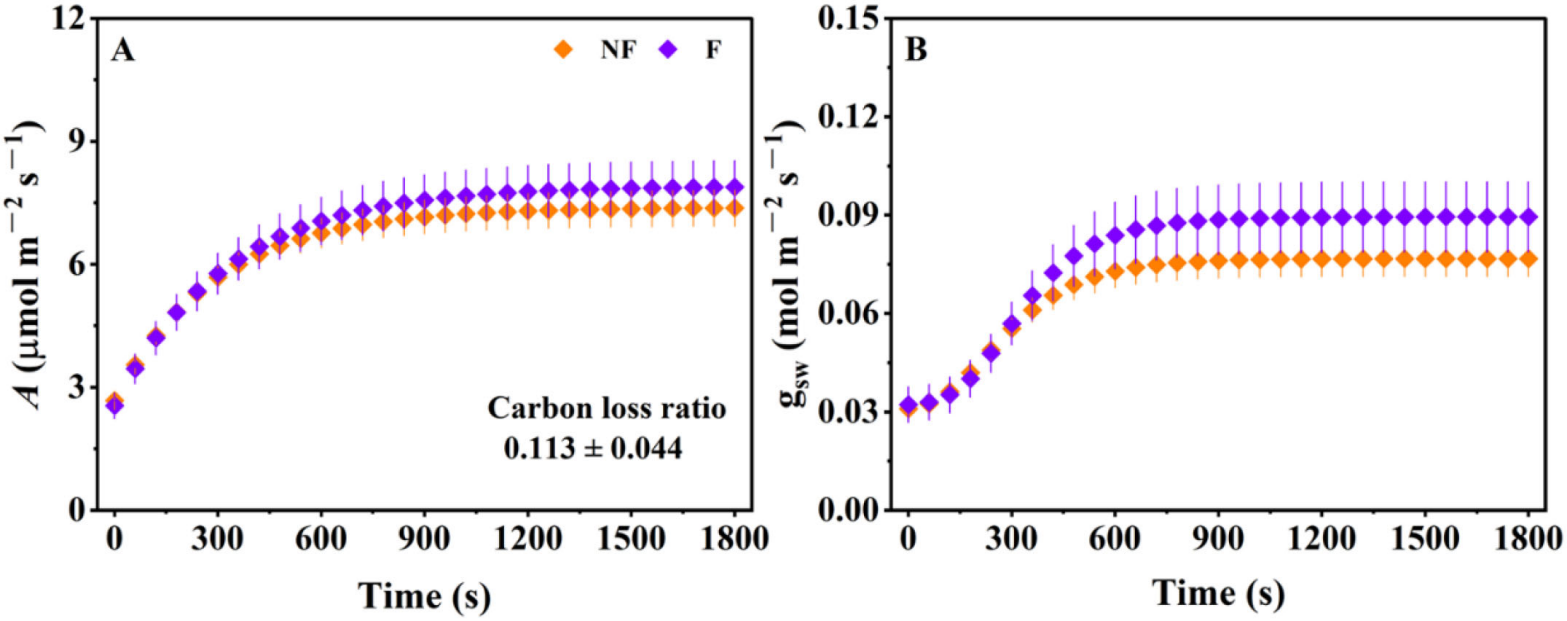
Photosynthetic traits during the photosynthetic induction period with and without fluorescence flashes. **(A)** Response of net photosynthetic rate **(A)** to a low-to-high light transition; **(B)** Response of stomatal conductance to water vapor during the light transition. Data are mean ± SE (n = 4).

**Figure 2:**
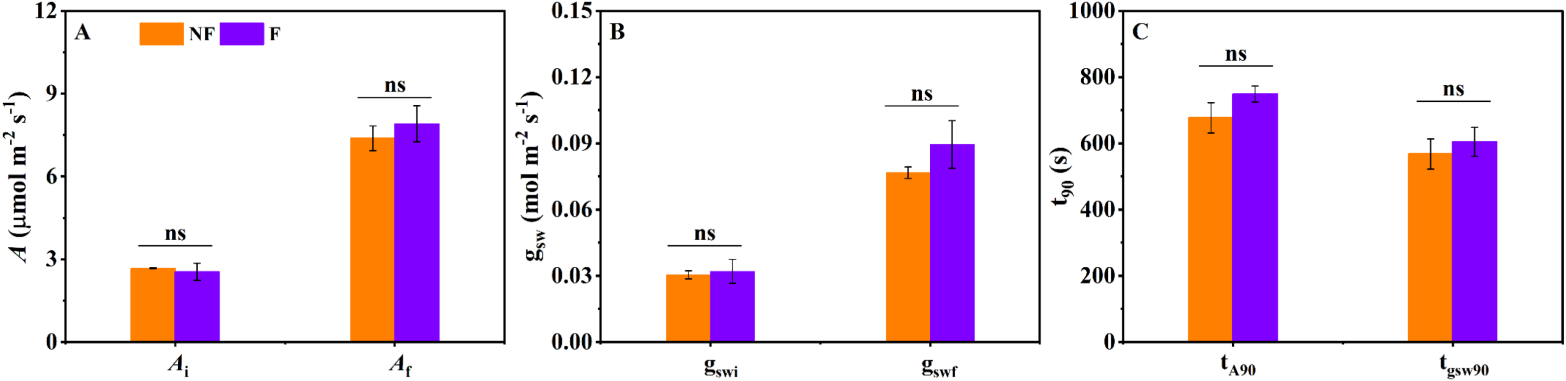
Photosynthetic induction characteristics derived from changes in net photosynthetic rate **(A)** and stomatal conductance to water vapor (g_sw_). **(A)** The initial *A* under low light (*A*_i_) and the final *A* under high light (*A*_f_); **(B)** The initial g_sw_ under low light (g_swi_) and the final g_sw_ under high light (g_swf_); **(C)** Time required for *A* (t_A90_) and g_sw_ (t_gsw90_) to reach 90% of their induction state. ns indicates no significant difference in photosynthetic traits between treatments with and without a fluorescence flash. Data are presented as mean ± SE (n = 4).

**Figure 1** shows the dynamic changes in *A* and g_sw_ in *Ficus carica* under a low-to-high light transition. In treatments with and without a fluorescence flash, net photosynthetic rate increased progressively and reached a steady-state (**Figure 1a**). Because photosynthesis increases progressively rather than instantaneously during the induction phase, the resulting delay led to a 11.3% reduction in potential carbon assimilation relative to an idealized condition where the photosynthetic rate immediately reaches its steady state. Similarly, g_sw_ exhibited a gradual increase during photosynthetic induction until reaching a stable level in both treatments (**Figure 1b**). As described in the Materials and Methods, to assess whether the saturation flashes affected the photosynthetic induction process, we compare *A* and g_s_ under low light (*A*_i,_ g_swi_), as well as final *A*, g_sw_, under high light (e.g. *A*_f_, g_swf_), and their response speed during photosynthetic induction. As illustrated in **Figure 1**, the temporal dynamics of CO_2_ assimilation and stomatal conductance during induction were highly comparable between treatments with and without saturation flashes. Specifically, *A*_i_ was 2.55 ± 0.31 and 2.68 ± 0.021 µmol m^-^² s^-^¹, respectively (**Figure 2a**). The *A*_f_ reached 7.91 ± 0.65 and 7.39 ± 0.45 µmol m^-^² s^-^¹ (**Figure 2a**), while the time required to achieve 90% of maximum *A* (t_A90_) was 12.5 ± 0.40 and 11.3 ± 0.77 min (**Figure 2c**). A similar pattern was observed for stomatal conductance: g_swi_ was 0.032 ± 0.005 and 0.031 ± 0.002 mol m^-^² s^-^¹, g_swf_ was 0.090 ± 0.011 and 0.077 ± 0.003 mol m^-^² s^-^¹, and t_gsw90_ was 10.1 ± 0.73 and 9.5 ± 0.76 min for the two treatments (**Figures 2b, c**). Statistical analyses revealed no significant differences across any of the six parameters, demonstrating that the saturation flashes used during fluorescence measurements did not alter the photosynthetic induction process. Moreover, the close alignment between the temporal trajectories of *A* and g_s_ in *Ficus carica* display remarkable alignment, with their nearly synchronized progression toward steady state suggesting a harmonized adjustment in carbon assimilation and stomatal behavior during the induction period.

### 3.2 Parameter-dependent variation in g_m_ and *V_cmax_* during photosynthetic induction

**Figure 3:**
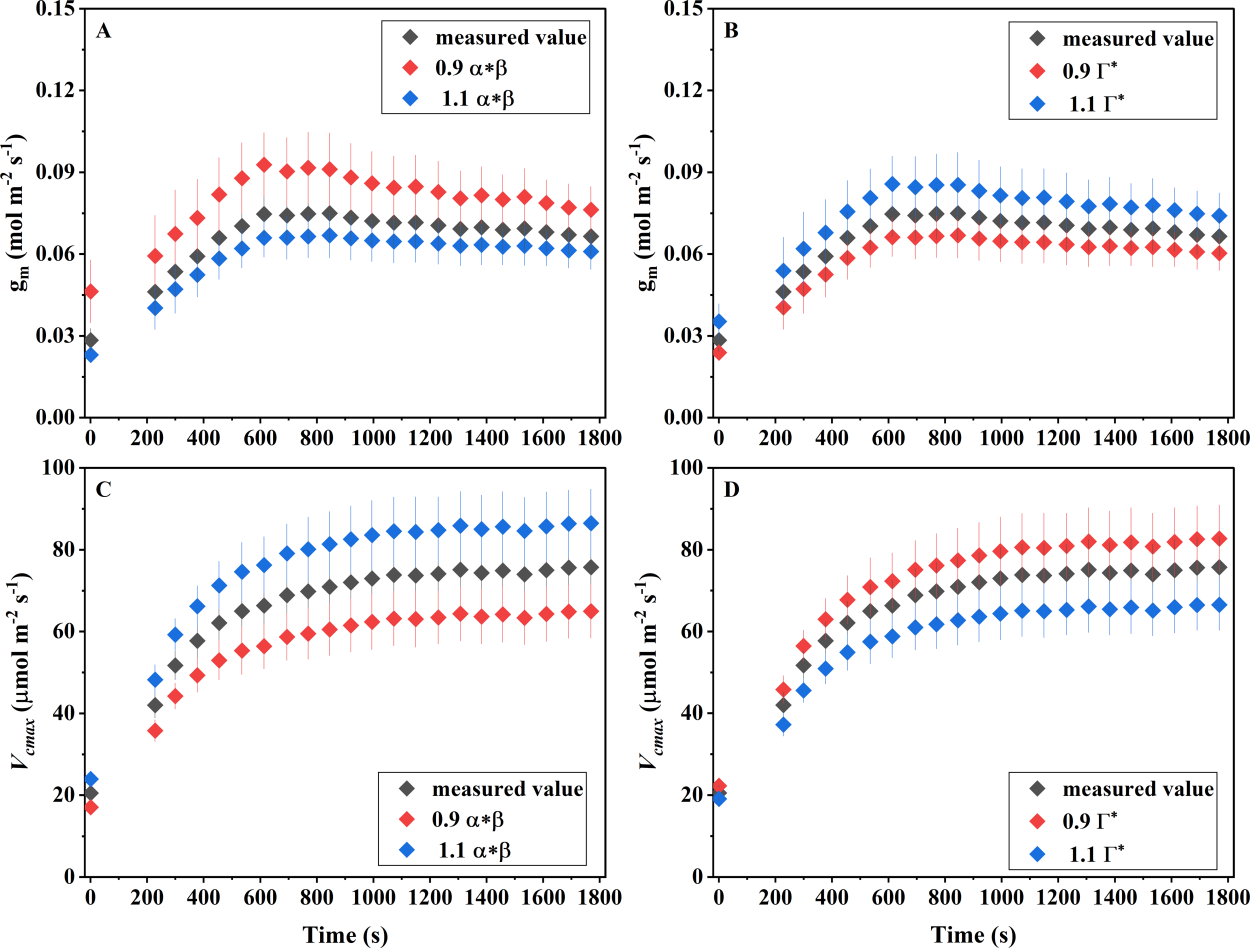
Sensitivity analysis of mesophyll conductance (gm) and maximum carboxylation rate (*Vcmax*) during the photosynthetic induction period to α, β, and the CO_₂_ compensation point in the absence of respiration (Γ*). **(A)** Sensitivity of gm to α and β; **(B)** Sensitivity of gm to Γ*; **(C)** Sensitivity of *Vcmax* to α and β; **(D)** Sensitivity of *Vcmax* to Γ*. α represents leaf absorptance and β denotes the partitioning of absorbed quanta between PSII and PSI. Data are presented as mean ± SE (n = 4).

The finding that saturation flashes do not affect the photosynthetic induction process (Section 2.1) establishes an essential prerequisite for applying the chlorophyll fluorescence method to estimate g_m_. With this prerequisite confirmed, we proceeded to the second step, which concerns the parameter‐correction requirements for g_m_ calculations. As shown in **Figures 3a, b**, when g_m_ was calculated using the measured values of αβ and Γ^*^, *Ficus carica* exhibited a conductance of 0.0284 ± 0.0043 mol m^-^² s^-^¹ under low light. During photosynthetic induction, g_m_ progressively increased and eventually stabilized at 0.067 ± 0.0072 mol m^-^² s^-^¹. As shown in **Figures 3c, d**, *V_cmax_* showed a comparable pattern, rising from 20.5 ± 1.36 to 75.7 ± 7.44 µmol m^-^² s^-^¹ from low light to the steady state.

Sensitivity analyses indicated that variations in both αβ and Γ^*^ markedly influenced g_m_ estimates. When αβ was reduced to 90% of its measured value (equivalent to lowering the electron transport–related term in the g_m_ calculation to 90%), the estimated g_m_ was higher than the actual value, increasing by 62.9% under low light and by an average of 20.1% under high light. In contrast, increasing αβ to 110% resulted in underestimation of g_m_, with reductions of 18.9% under low light and 10.2% under high light (**Figure 3c**). Changes in Γ^*^ produced the opposite effect. Reducing Γ^*^ to 90% of its measured value led to underestimation of g_m_, with decreases of 15.9% under low light and 10.5% on average under high light. Increasing Γ^*^ to 110% caused overestimation of g_m_, with increases of 24.2% under low light and 13.4% under high light (**Figure 3d**). These results collectively indicate that g_m_ estimates were more sensitive to parameter variation under low light than under high light.

Because *V_cmax_* depends on chloroplastic CO_2_ concentration (C_c_) (Eqns 9 and 10), and changes in g_m_ alter C_c_, variations in αβ and Γ^*^ indirectly affected *V_cmax_* as well. When αβ was reduced to 90%, *V_cmax_* decreased by 16.8% under low light, and it decreased by 14.5% under high light. Increasing αβ to 110% led to overestimation of *V_cmax_*, with increases of 20.8% under low light and 2.3% under high light. Conversely, decreasing Γ^*^ to 90% resulted in overestimation of *V_cmax_*, with an increase of 8.5% for low light and 9.1% for high light, whereas increasing Γ^*^ to 110% caused underestimation of *V_cmax_*, with a decrease of 7.0% for low light and 17.8% for high light.

**Figure 4:**
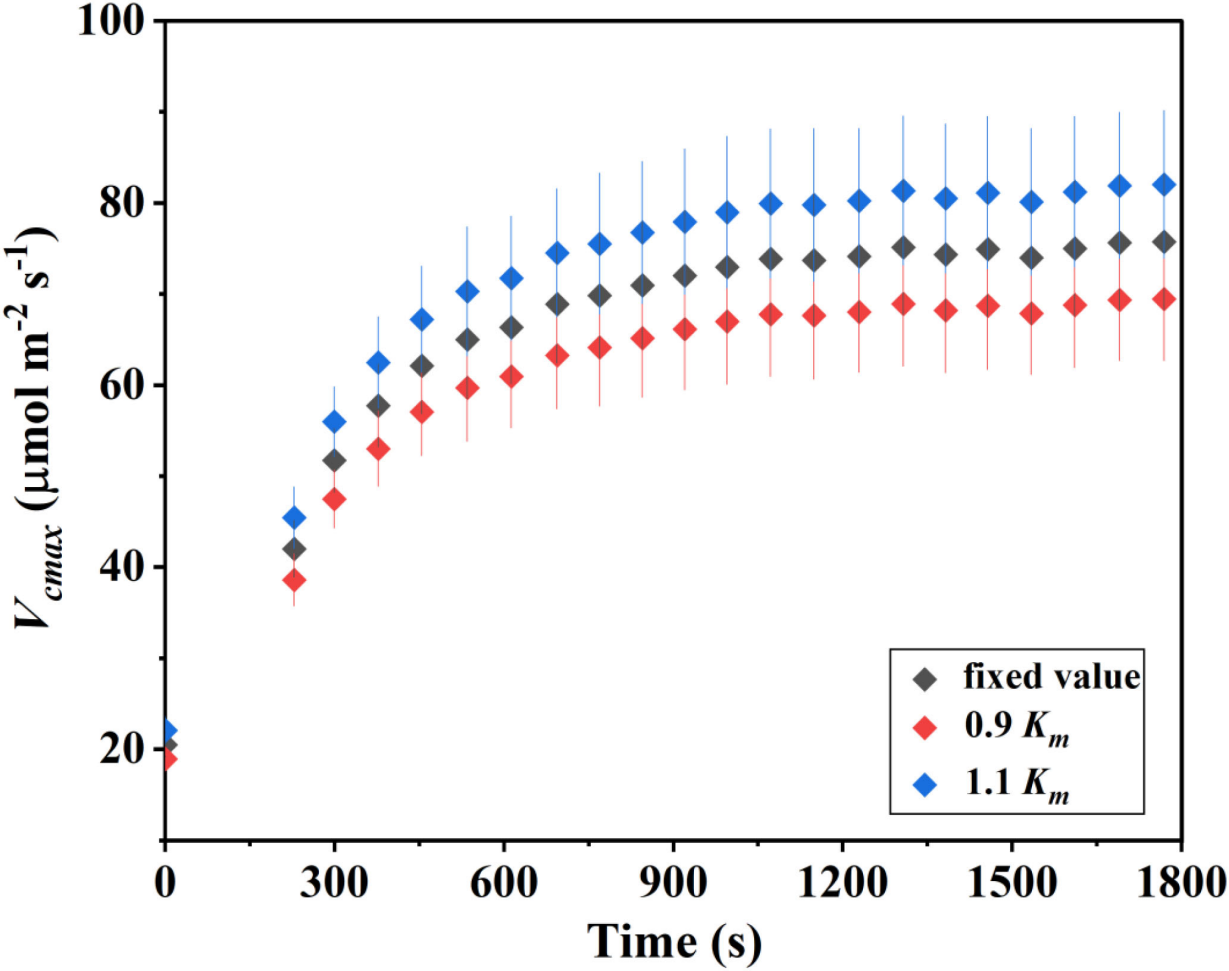
Sensitivity analysis of the maximum carboxylation rate (*V_cmax_*) to the Michaelis–Menten constant of Rubisco (*K_m_*) during the photosynthetic induction period. The values for *K*_m_ was fixed at 541.9 μmol mol^-1^, based on Walker et al. (2013), with a 10% variation applied during the sensitivity analysis. Data are presented as mean ± SE (n = 4).

In addition to the sensitivity of g_m_ and *V_cmax_* to variations in αβ and Γ^*^, we also evaluated the impact of the Rubisco affinity constant (*K_m_*) on *V_cmax_* estimation. As shown in **Figure 4**, changes in *K_m_* had a comparatively smaller influence. When *K_m_* was reduced to 0.9 *K_m_* or increased to 1.1 *K_m_*, the resulting variation in *V_cmax_* was approximately 8.2%. This effect size was notably lower than that observed for αβ and Γ^*^, indicating that uncertainties in *K_m_* contribute less to the overall variability of *V_cmax_* during photosynthetic induction.

### 3.3 Influence of αβ, Γ^*^, and *K_m_* variations on photosynthetic limitation partition

**Figure 5:**
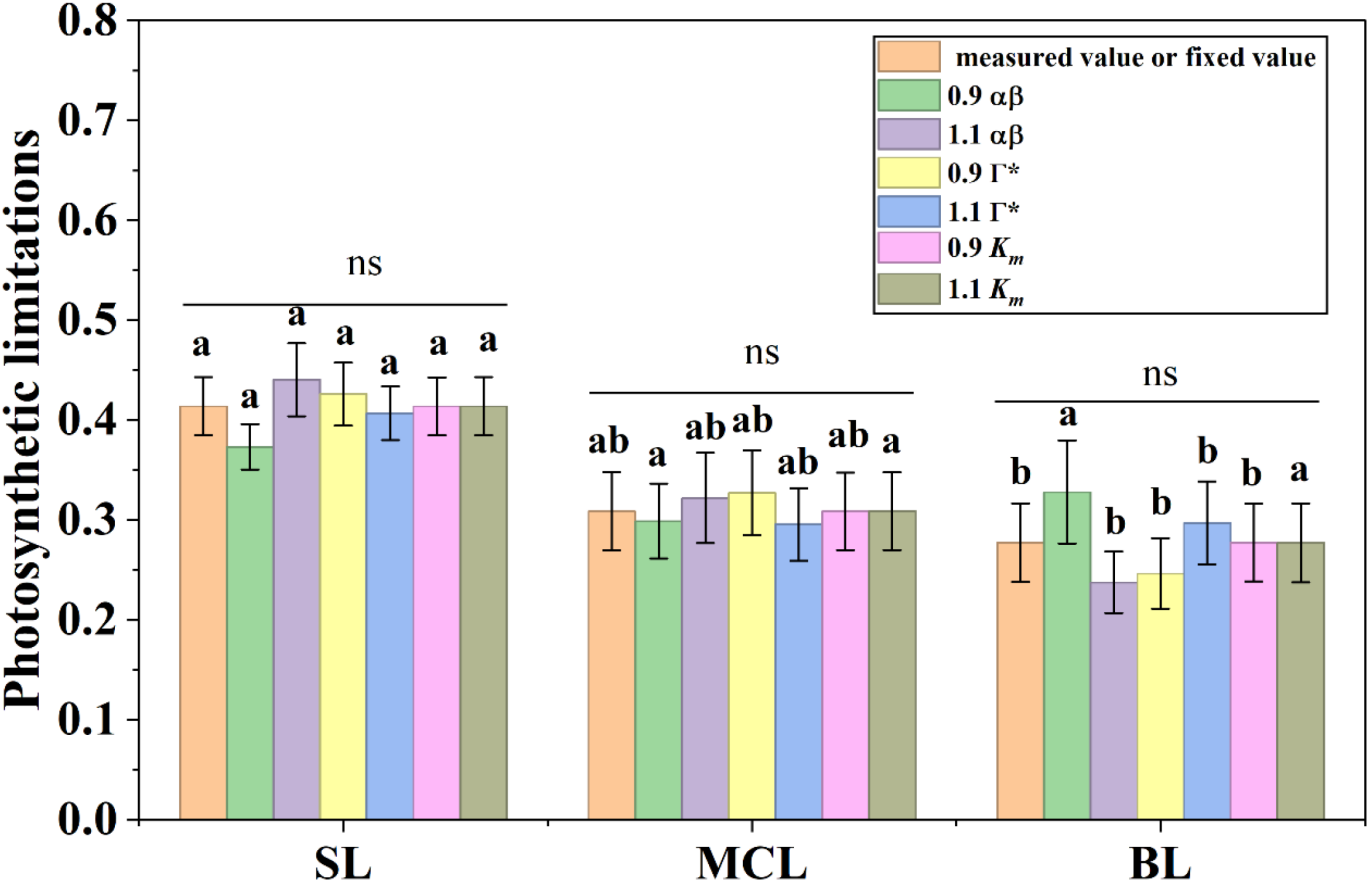
Dynamic Sensitivity analysis of photosynthetic limitations to αβ, the CO_2_ compensation point in the absence of respiration (Γ^*^), and the Michaelis–Menten constant of Rubisco (*K_m_*). α represents leaf absorptance and β denotes the partitioning of absorbed quanta between PSII and PSI. The values for α and β were experimentally measured, while *K*_m_ was fixed at 541.9 μmol mol^-1^, based on Walker et al. (2013), with a 10% variation applied during the sensitivity analysis. Data are presented as mean ± SE (n = 4). Different letters indicate significant differences among stomatal limitation (SL), mesophyll conductance limitation (MCL), and biochemical limitation (BL) under each sensitivity analysis scenario. ns indicates no significant difference in photosynthetic limitations among sensitivity analysis scenarios.

Based on the changes in *A*, g_s_, g_m_, and *V_cmax_* during photosynthetic induction, we quantified the relative contributions of each limitation component, as shown in **Figure 5**. Stomatal limitation, mesophyll conductance limitation, and biochemical limitation accounted for 41.4%, 30.9%, and 27.7% of the total limitation, respectively. These values indicate that stomatal limitation was the primary constraint on photosynthetic induction in *Ficus carica*, although it was not statistically different from mesophyll conductance limitation.

We subsequently assessed the effects of variations in αβ, Γ^*^, and *K_m_* on the limitation partitioning. When αβ was reduced to 90% of its measured value, SL and MCL decreased by 9.5% and 2.1%, whereas BL increased by 17.7%. Increasing αβ to 110% produced the opposite pattern, with SL and MCL increasing by 6.2% and 3.9%, accompanied by a 13.9% reduction in BL. Reducing Γ^*^ to 90% of its measured value increased SL and MCL by 2.9% and 5.9%, while BL decreased by 11.2%. Conversely, increasing Γ^*^ to 110% led to reductions of 1.5% and 3.9% in SL and MCL, and a corresponding 7.3% increase in BL. Although these parameter adjustments changed the numerical values of individual limitation components, statistical analyses showed no significant differences compared with those calculated using the observed parameter values. In contrast, altering *K_m_* had negligible effects, as SL, MCL, and BL remained essentially unchanged. Taken together, these findings demonstrate that variations in αβ, Γ^*^, and *K_m_* do not alter the overall limitation pattern during photosynthetic induction in *Ficus carica*.

## 4 Discussion

### 4.1 Effect of saturation flashes on the photosynthetic induction

Quantifying the real-time dynamics of g_m_ during photosynthetic induction is essential for elucidating the regulatory mechanisms underlying photosynthetic efficiency under fluctuating light. In recent years, chlorophyll fluorescence–based approaches have been increasingly employed to estimate g_m_ during induction (Sun et al., 2022a, Sun et al., 2022b; Sun et al., 2023; Wang et al., 2023). This methodological expansion has largely been driven by the growing recognition that g_m_ represents a key limitation in photosynthetic efficiency during induction period (Liu et al., 2022; Pan et al., 2025). Consequently, accurately capturing kinetics of g_m_ is critical for understanding whole-plant productivity and carbon gain under realistic conditions. However, the saturation flashes applied during fluorescence measurements typically reach intensities of up to approximately 8000 μmol m^-^² s^-^¹ (Sun et al., 2022a, Sun et al., 2022b; Sun et al., 2023; Wang et al., 2023), raising the concern that they may perturb the photosynthetic state of leaves and thereby alter the natural course of photosynthetic induction. It is therefore necessary to determine whether these flashes influence the physiological induction process. Our results (**Figures 1**, **2**) show that key photosynthetic parameters exhibit no significant differences between treatments with and without saturation flashes, indicating that the flashes do not measurably affect the intrinsic induction dynamics. This lack of disturbance is particularly important because saturation flashes are used repeatedly within a short time window, and any photoinhibitory or regulatory side effects would otherwise accumulate across the induction period. The convergence of *A*, and g_sw_ between treatments therefore provides robust evidence that the fluorescence technique is compatible with transient-state measurements. These findings suggest that chlorophyll fluorescence techniques are methodologically suitable for monitoring g_m_ in real time during photosynthetic induction. Thus, researchers can confidently adopt fluorescence-based g_m_ estimation in fluctuating-light studies without concern for measurement-induced artifacts. Although not significant, saturation flashes indeed overestimated both *A* and g_s_ (**Figures 1**, **5**) compared with ordinary gas exchange measurements. Future studies could collect more data to analyze the empirical relationships of photosynthetic parameters with and without saturation flashes, allowing for potential calibration, thereby enhancing the reliability of fluorescence-based g_m_ estimation.

### 4.2 Dominance of stomatal limitation in *Ficus carica* during photosynthetic induction as the primary constraint on photosynthetic induction

Building on the confirmation that the fluorescence-based method is fundamentally reliable, we conducted a photosynthetic limitation analysis using *A*, g_s_, g_m_, and *V_cmax_* obtained from combined chlorophyll fluorescence and gas-exchange measurements. Our results show that photosynthetic induction led to a carbon loss of 11.3% (**Figure 1**). This loss is lower than reported in several previous studies (Taylor and Long, 2017; Lawson and Blatt, 2014), which may be attributed to the relatively synchronized responses of *A* and g_s_ during induction, as reflected by the similar values of t_A90_ and t_gsW90_ (**Figure 2**). Such synchronization suggests that mesophyll and stomatal processes are co-regulated in *Ficus carica* to a greater extent than in many other species, where delayed stomatal opening often imposes a substantial bottleneck on CO_2_ diffusion (Taylor and Long, 2017; Lawson and Blatt, 2014; McAusland et al., 2016). Time-integrated limitation analysis further revealed that stomatal conductance represents the primary limiting factor during induction in *Ficus carica* (**Figure 5**). Previous study has shown that variation in stomatal limitation among species or treatments is often associated with differences in stomatal response time—slower responses generally lead to stronger stomatal limitation (Deans et al., 2019a). Additionally, the magnitude of the gs shift from low to high light also affects the extent of stomatal limitation (Deans et al., 2019b), which is consistent with our findings. In species where gs remains dramatically lower under low light, the requirement for large-scale opening following high-light transitions exacerbates both the duration and magnitude of stomatal limitation (Liu et al., 2022). *Ficus carica* appears to partially avoid this by maintaining moderately high g_s_ under low light relative to its g_s_ under high light condition, reducing the induction burden. These observations suggest that improving photosynthetic induction efficiency requires both accelerating the dynamic responses of key photosynthetic factors, such as g_s_, and reducing the disparity in physiological states between low- and high-light conditions. Such evidence supports the notion that constitutively higher baseline g_s_, or faster signaling pathways for stomatal adjustment, could serve as breeding or biotechnology targets for improving carbon gain under fluctuating light. Importantly, in our case, excluding g_m_ as an independent limiting factor would cause its contribution to be absorbed into the biochemical limitation component, leading to the incorrect conclusion that biochemical limitation exceeds stomatal limitation in *Ficus carica*. This outcome reinforces a critical methodological point: g_m_ must be explicitly considered when assessing photosynthetic limitations during induction (Liu et al., 2022). Neglecting g_m_ may bias not only the magnitude but also the interpretation of the dominant limitation type, highlighting the need for complete diffusion pathway analyses in photosynthetic induction.

### 4.3 Role of parameter sensitivity in photosynthetic induction analyses

Given the critical role of g_m_ in understanding photosynthetic induction, accurately quantifying g_m_ during the induction process is essential. Nevertheless, many studies adopt fixed parameter values—such as αβ = 0.42 and Γ^*^ = 40 μmol mol^-^¹—without evaluating their potential impact (Sun et al., 2022a, Sun et al., 2022b; Sun et al., 2023; Wang et al., 2023). To address this issue, we performed a sensitivity analysis of g_m_ to these commonly used parameters. Our results show that overestimating αβ leads to an underestimation of g_m_, whereas underestimating αβ results in higher g_m_ values. Conversely, overestimating Γ^*^ causes g_m_ to be overestimated (**Figure 3b**). This parameter-dependent variability indicates that even small deviations from true physiological values can propagate into substantial g_m_ uncertainties, especially under non-steady-state conditions where rapid flux changes amplify calculation sensitivity, which is consistent with the results found in the steady-state light conditions (Xiong et al., 2015). Importantly, we further examined how variations in g_m_ induced by parameter assumptions influence the estimation of *V_cmax_*. Our analysis indicates that *V_cmax_* changes substantially with alterations in αβ, Γ^*^, and the resulting g_m_. Additionally, *K_m_* strongly affected *V_cmax_*: a 10% change in *K_m_* resulted in an approximately 8% change in *V_cmax_*, underscoring its importance for biochemical parameterization (**Figure 4**). This tight coupling suggests that any mis-parameterization can distort not only diffusional components but also biochemical interpretations, potentially leading to erroneous conclusions about enzyme kinetics or Rubisco activation during induction.

Although these parameters substantially influence g_m_ and *V_cmax_* estimates, our primary concern was whether such changes would alter the outcomes of photosynthetic limitation analyses. Our results demonstrate that these parameter variations do not affect the qualitative conclusions of limitation analysis (**Figure 5**). To our best knowledge, this is the first systematic assessment of how variations in αβ, Γ^*^, and *K_m_* affect g_m_, *V_cmax_*, and the resulting limitation partitioning. This robustness implies that, although absolute g_m_ and *V_cmax_* values shift with parameter choices, the relative contributions of stomatal, mesophyll, and biochemical limitations remain stable, thereby preserving the biological interpretation. It should be noted that precise calibration of these parameters is labor-intensive. Determining αβ requires low-O_2_ light response curves (Yin et al., 2009), while Γ^*^ estimation relies on CO_2_ response curves under multiple light levels (Brooks and Farquhar, 1985). Accurate quantification of *K_m_* often requires radioactive ¹^4^C-based assays (Walker et al., 2013). Given these technical challenges, if g_m_ remains positive without parameter correction, calibration may not be necessary. However, when g_m_ becomes negative, parameter correction is indispensable. Thus, for routine studies where g_m_ is consistently positive and limitation analysis is the primary goal, fixed parameter assumptions may be acceptable, but for mechanistic or species-comparison studies, full parameterization remains essential for accurate physiological interpretation.

### 4.4 Limitations and future directions for fluorescence-based g_m_ estimation

We acknowledge that the reliability of fluorescence-based g_m_ estimation during photosynthetic induction could be influenced by factors such as alternative electron transport, the stoichiometry of ATP/NADPH production, chloroplast movement altering leaf absorbance, and mitochondrial respiration under light conditions (Kaiser et al., 2017). However, as demonstrated by Pan et al. (2025), key parameters α and β did not differ significantly between the induction phase and steady-state conditions, suggesting minimal variation in light absorption. Furthermore, Pan et al. (2025) validated the fluorescence method against stable isotope techniques during induction, confirming its feasibility—at least in cotton. It should be noted that method performance may be species-specific; for instance, Kaiser et al. (2017) reported lower reliability in tomato during early induction. Our sensitivity analysis aligns with this, showing that g_m_ estimates are most sensitive to input parameters under low light (**Figures 3a, b**). Importantly, the sensitivity of *V_cmax_* stems from deviations in C^c^ derived from g_m_; thus, improving the reliability of g_m_ estimation directly addresses uncertainties in *V_cmax_* calculation. While this study offers a valuable proof of concept, its conclusions are necessarily constrained by the limited dataset (n=4 leaves from a single species under one set of growth conditions). This limits the generalizability of findings related to limitation partitioning and parameter robustness. Future work employing larger sample sizes across diverse species and environments, coupled with direct validation (e.g., via stable isotopes), will be essential to establish the broader applicability of the fluorescence method for g_m_ estimation during photosynthetic induction.

## 5 Conclusions

In summary, our study provides a comprehensive assessment of the methodological reliability and physiological implications of chlorophyll fluorescence–based approaches for evaluating photosynthetic induction, specifically in *Ficus carica* seedlings under the given environmental conditions. By confirming that saturation flashes do not alter the intrinsic induction dynamics, we establish a robust foundation for using fluorescence techniques to monitor g_m_ in fluctuating-light environments, though the conclusions are primarily applicable to this species, developmental stage, and growth condition. Furthermore, our limitation analysis reveals that stomatal conductance constitutes the primary constraint during induction in *Ficus carica*, emphasizing the importance of improving stomatal responsiveness and minimizing low- to high-light disparities to enhance carbon gain under dynamic light conditions. Importantly, we demonstrate that g_m_ and *V_cmax_* are sensitive to commonly assumed biochemical parameters (αβ, Γ^*^, and *K_m_*), yet the overall outcomes of photosynthetic limitation partitioning remain stable despite these variations. To our knowledge, this represents the first systematic evaluation of how parameter uncertainty propagates through g_m_ estimation, *V_cmax_* calculation, and limitation analysis. Collectively, these findings highlight the necessity of carefully considering diffusion and biochemical parameters while also providing reassurance that core biological interpretations remain robust, thereby offering important methodological guidance for future studies on photosynthetic induction.

## Data Availability

The original contributions presented in the study are included in the article/supplementary material. Further inquiries can be directed to the corresponding authors.
